# A patient with hereditary transthyretin amyloidosis involving multiple cranial nerves due to a rare p.(Phe84Ser) variant

**DOI:** 10.1515/tnsci-2022-0219

**Published:** 2022-06-07

**Authors:** Xian-rang Yan, Ming-fan Hong, Zhi-hua Zhou, Ai-qun Liu, Zhong-xing Peng, Wei-feng Wu, Cheng Jing, Jia-xiu Lin, Ying Long, Qing-yun Yu

**Affiliations:** Department of Neurology. The First Affiliated Hospital of Guangdong Pharmaceutical University, No. 19, Nonglinxia Road, Yuexiu District, Guangzhou 510000, China

**Keywords:** amyloid polyneuropathy, familial amyloidosis peripheral disease, transthyretin, neuroelectrophysiology, p.(Phe64Ser), p.(Phe84Ser)

## Abstract

We report a 30-year-old man involving gastrointestinal symptoms, vitreous opacity, and multiple cranial neuropathies. Transthyretin-related hereditary amyloidosis genetic testing revealed a rare c.251T > C variant p.(Phe84Ser). Only four cases with this variant have been reported before.

We report a 30-year-old man involving gastrointestinal symptoms, vitreous opacity, and multiple cranial neuropathies. Transthyretin (TTR)-related hereditary amyloidosis (ATTRv, v for variant) [1] genetic testing revealed a rare c.251T > C variant p.(Phe84Ser). Only four cases with this variant have been reported before.

The patient was a 30-year-old Han Chinese male from Jieyang, Guangdong Province, China; he had been 28 years old at the time of onset. He initially presented with gastrointestinal symptoms such as abdominal distension, alternating diarrhea and constipation, and the sensation that his swallowing was obstructed, and he visited the Department of Gastroenterology several times. No abnormalities were observed by gastrointestinal endoscopy, and a barium swallow did not reveal any esophageal abnormalities. One year later, he developed limb numbness in the extremities, saw more “floaters,” or small specks, in front of both eyes, and had decreased visual acuity accompanied by symptoms of autonomic dysfunction such as erectile dysfunction. As the disease progressed, his muscle strength decreased, he developed a limp, and his vision deteriorated significantly before other examinations were performed. The patient had lost 10 kg since the onset of symptoms. The patient had previously been healthy and had no history of taking drugs.

In terms of family history, the patient’s mother, who died of unclear causes, had also developed decreased visual acuity at approximately 35 years of age; her family history was unknown. The patient’s sister had gradually developed decreased visual acuity 2 years ago, when she was 22 years old, and later died of accidental drowning due to blindness in both eyes. The patient was married and had three children (two male and one female), who did not present with the above symptoms; his father was also healthy (the pedigree is shown in Figure 1). To date, none of these family members have been tested for the *TTR* genes.

**Figure 1:**
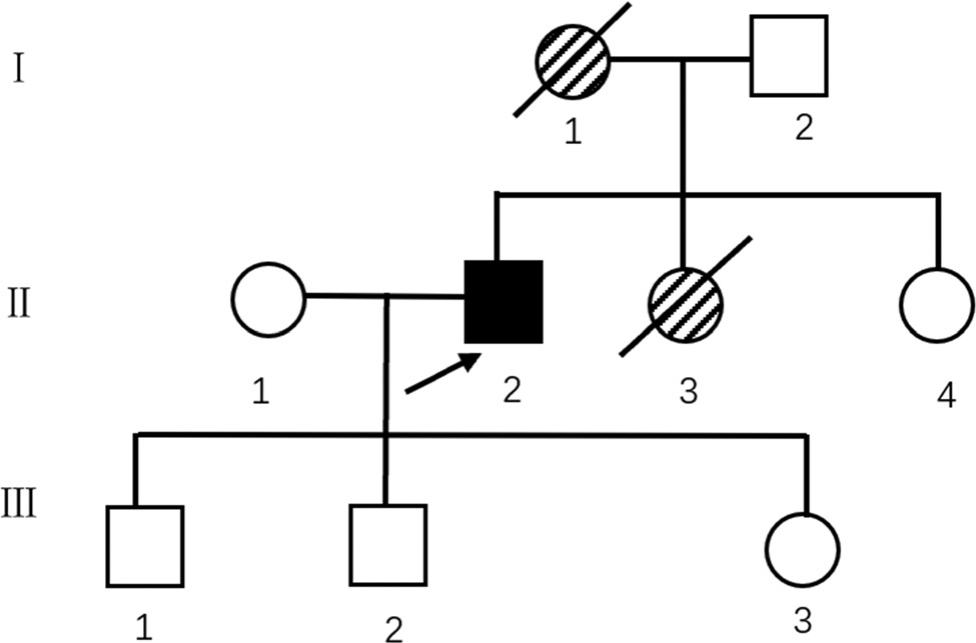
Family pedigree depicting the propositus (Ⅱ2) and displaying his sister (Ⅱ3) and mother (Ⅰ1), who had the same symptoms but not confirmed by genetic testing. Roman numbers refer to generations.

On physical examination, the patient was noted to be a weak-appearing and anxious male. He had postural hypotension (blood pressure 92/56 mmHg standing, 140/103 mmHg sitting). His speech was unclear and contained few words. The bilateral pupils were equal, round, at 6.0 mm, and had a sensitive light reflex. The examination also found a moderate degree of bilateral blepharoptosis (upper eyelid covers 4 mm of the limbus, and the binocular fatigue test is negative), a rightward deviation of the tongue on protraction, a leftward deviation of the uvula, a loss of the gag reflex of soft palate, and no lingual tremor. Auscultation did not reveal any abnormal breath sounds. The abdomen was soft without tenderness, and there was no hepatosplenomegaly. The proximal muscles of the limbs were significantly atrophied with weakness of the limbs, the muscle tension of the limbs was normal, the tendon reflexes were diminished, the sensory system was normal, and the bilateral Babinski signs were negative.

Ocular examination showed posterior subcapsular mild opacity, severely vitreous cotton-wool opacity, and invisible fundus in the right eye; moderate vitreous cotton-wool opacity in the left eye; and no hemorrhagic foci or exudative foci in the fundus (ultrasonography of both eyes is shown in Figure 2).

**Figure 2:**
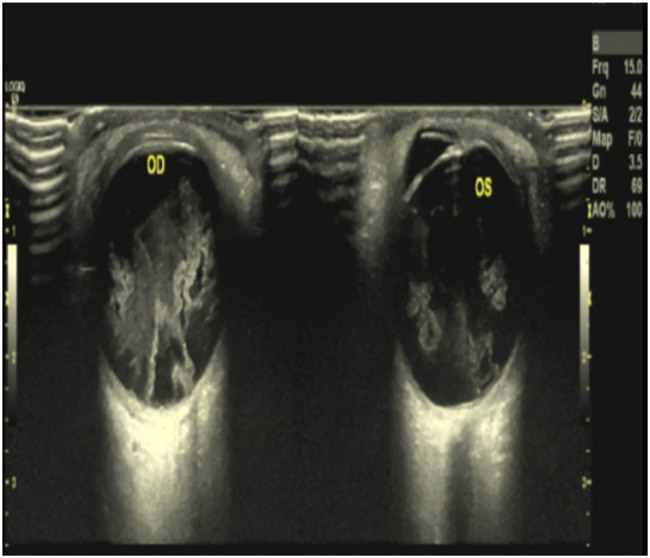
Ultrasonography of both eyes revealed vitreous opacity in both eyes.

Routine blood examination revealed mildly decreased hemoglobin (11 g/dL). Cerebrospinal fluid analysis revealed normocytosis with an increased protein concentration of 141.2 mg/dL. Electrocardiography revealed sinus rhythm, low limb voltage, and a poor R wave increment in the left anterior wall (electrocardiography is shown in Figure 3). Brain and spinal cord magnetic resonance imaging were normal without leptomeningeal enhancement.

**Figure 3:**
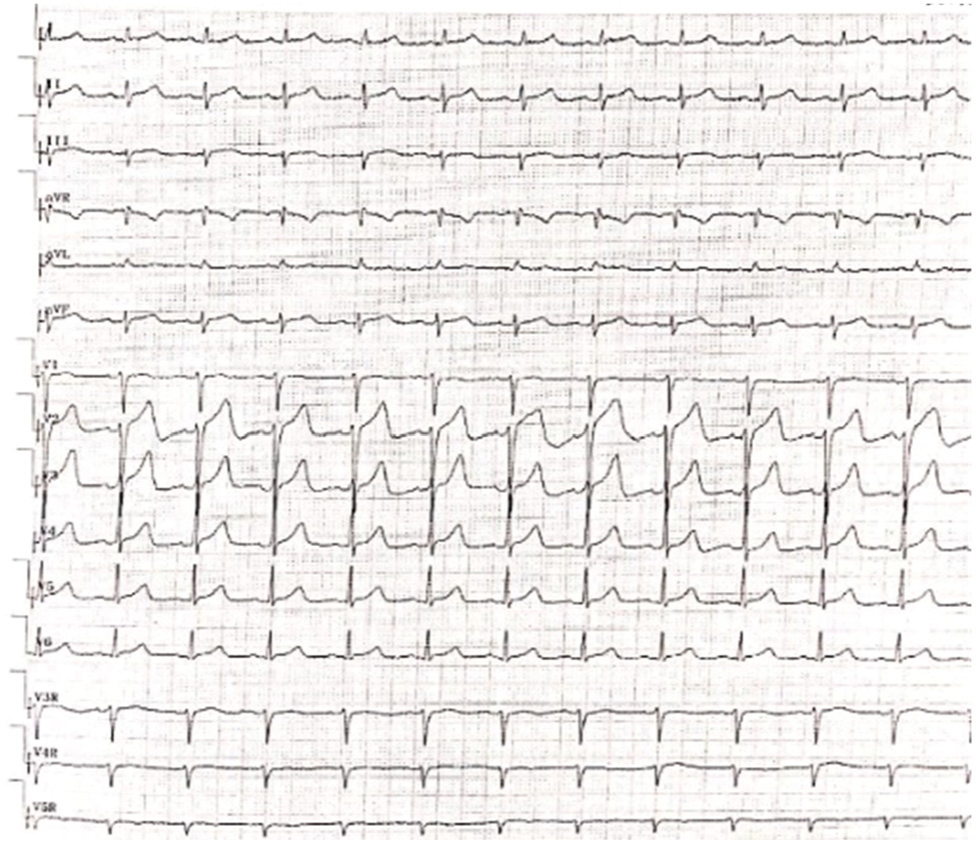
Electrocardiography revealed sinus rhythm, low limb voltage, and a poor R wave increment in the left anterior wall.

Electrophysiological examination revealed a wide range of nerve involvement; symmetrical sensory and motor nerves around the extremities were involved, and the axonal injury was prominent, in line with length-dependent characteristics. Additionally, the F wave latency was prolonged, and the output rate was decreased. Multiple cranial nerves were also affected: the oculomotor nerve, the facial nerve (prolonged motor latency, significantly reduced amplitude), the auditory nerve (prolonged brainstem auditory evoked potentials I wave latency), and the optic nerve (prolonged visual evoked potentials latency) (Figure 4) were involved. After Sanger sequencing of the *TTR* gene, a heterozygous c.251 T > C variant (p.(Phe84Ser), previously known as p.(Phe64Ser)) was revealed. (The signal region encoded by the first 20 amino acids of TTR was omitted previously because the residues in this portion do not take place in the mature peptide. Nonetheless, the present nomenclature requires that the first nucleotide of the start codon, that is, “c.1,” and “ATG” encoding “methionine” is p.1. For this reason, the change written as “p.Phe64Ser” in the past is now “p.(Phe84Ser).”) (Figure 5).

**Figure 4:**
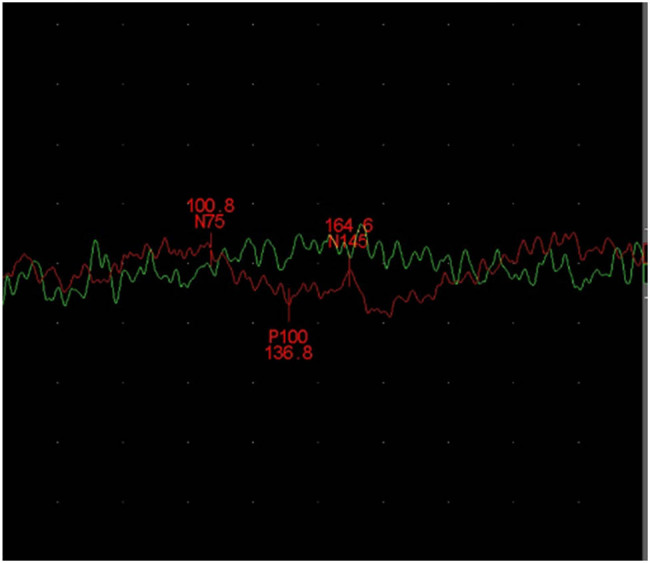
The visual evoked potentials latency was prolonged.

**Figure 5:**
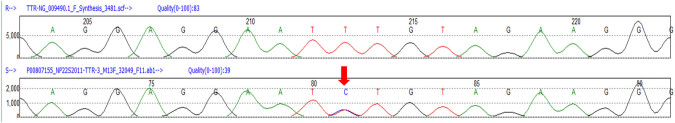
Electropherogram of the *TTR* gene sequencing is shown: (Top) Sequencing diagram of the healthy control, and (bottom) points with the red-colored arrow the c.251T＞C (p.(Phe84Ser)) alteration in the patient(II:2).

The reporting of this study conforms to the CASE guidelines. Written consent was obtained from the patient. The study was approved by the Ethics Committee of The First Affiliated Hospital of Guangdong Pharmaceutical University (Guangzhou, Guangdong, China) (No. 2022-14) and adhered to the tenets of the Declaration of Helsinki.

This patient was diagnosed with TTR p.(Phe84Ser) amyloid peripheral neuropathy by genetic testing. ATTRv has been reported to have a global distribution, with 140 different mutations now have been reported, and its clinical presentation is varied [2]. However, the TTR p.(Phe84Ser) variant and its clinical phenotypes is rarely reported. In 1988, p.(Phe84Ser) was first reported by Umeichi in three patients in a Canadian family with lesions in the lingual muscle, meninges, spinal cord, retina, vitreous, peripheral nerves, and internal organs; the main clinical manifestation was hemiplegia. Migraine, periodic coma, epilepsy, cerebral hemorrhage, myelopathy, visual impairment, deafness, and peripheral neuropathy were later confirmed by Umeichi et al. [3] to be associated with TTRv, of which the first reported example was the p.(Phe64Ser) variant. (P.S.: the change written as “p.Phe64Ser” in the past is now “p.(Phe84Ser)”). Naderi [4] reported a second case of the p.(Phe64Ser) variant (P.S.: the change written as “p.(Phe64Ser)” in the past is now “p.(Phe84Ser)”), first identified in an African-American family with clinical manifestations of severe gastrointestinal symptoms. Xin et al. [5] reported the first case of central nervous system involvement, such as pyramidal signs, ataxia signs, elevated cerebrospinal fluid protein, and meningeal enhancement, in a patient in China who had gastrointestinal symptoms. In 2021, Zhang et al. [6] presented a 26-year-old Chinese female with the p.(Phe64Ser) variant, which is the second case in China.

ATTRv usually presents as a length-dependent sensorimotor polyneuropathy [7,8], and the electrophysiological findings in this case also present typical neuropathic sensorimotor impairment. However, most previously reported cases analyzed the characteristics of spinal nerves, and further examination of cranial nerves was not performed to identify the presence or absence of damage. In the present case, the patient had symptoms such as loss of the pupil light reflex, ptosis, and loss of gag reflex with a right deviation of the tongue, but cranial magnetic resonance showed no significant abnormality. We performed an electrophysiological examination on this patient to test for the presence of multiple cranial neuropathies; the results strongly suggested that the p.(Phe84Ser) variant was associated with central nervous system involvement, just like the case reported by Naderi of an early-onset case with no family history, characterized by severe gastrointestinal symptoms, no evidence of central involvement, which may be due to the early onset of the disease, slow development of central nervous system impairment, or ethnic and geographical differences. In general, genetic variants that cause inherited diseases are closely related to many aspects of the phenotype, including incidence, geographical location, and progression. This patient, who is, to the best of our knowledge, the first patient with TTR Phe84Ser amyloidosis demonstrated by electrophysiological examination, had multiple cranial neuropathy, including the second, third, seventh, ninth, and tenth nerve palsy. There have been few other reports of cranial neuropathy associated with ATTRv in the literature. In this case, the patient had nonspecific lesions of multiple cranial nerves in addition to ocular symptoms, autonomic dysfunction, and multiple peripheral neuropathies, which were considered to be due to amyloid deposition. In summary, when multiple peripheral neuropathies occur, clinicians must not only check for a positive family history but not also perform necessary neurophysiological tests to facilitate early diagnosis and prompt treatment. In addition, the present case broadens our understanding of the possible presence of a very rare TTR amyloidosis genotype.
